# Treatment-Induced Neuropathy in Diabetes (TIND)—Developing a Disease Model in Type 1 Diabetic Rats

**DOI:** 10.3390/ijms22041571

**Published:** 2021-02-04

**Authors:** Petra Baum, Severin Koj, Nora Klöting, Matthias Blüher, Joseph Classen, Sabine Paeschke, Martin Gericke, Klaus V. Toyka, Marcin Nowicki, Joanna Kosacka

**Affiliations:** 1 Department of Neurology, University of Leipzig, Liebigstraße 20, D-04103 Leipzig, Germany; Petra.Baum@medizin.uni-leipzig.de (P.B.); Severin.Koj@medizin.uni-leipzig.de (S.K.); Joseph.Classen@medizin.uni-leipzig.de (J.C.); 2 Department of Medicine, University of Leipzig, Liebigstraße 21, D-04103 Leipzig, Germany; Nora.Kloeting@medizin.uni-leipzig.de (N.K.); Matthias.Blueher@medizin.uni-leipzig.de (M.B.); 3 Helmholtz Institute for Metabolic, Obesity and Vascular Research (HI-MAG), Helmholtz Zentrum München, University of Leipzig, Philipp-Rosenthal-Straße 27, D-04103 Leipzig, Germany; 4 Institute of Anatomy, University of Leipzig, Liebigstraße 13, D-04103 Leipzig, Germany; Sabine.Paeschke@medizin.uni-leipzig.de (S.P.); Marcin.Nowicki@medizin.uni-leipzig.de (M.N.); 5 Institute of Anatomy and Cell Biology, University of Halle, Große Steinstraße 52, D-06108 Halle, Germany; Martin.Gericke@medizin.uni-halle.de; 6 Department of Neurology, University of Würzburg, Josef-Schneider-Straße 11, D-97080 Würzburg, Germany; kv.toyka@uni-wuerzburg.de

**Keywords:** BB/OKL rats, peripheral neuropathy, sciatic nerve, TIND, Type 1 diabetes

## Abstract

Treatment-induced neuropathy in diabetes (TIND) is defined by the occurrence of an acute neuropathy within 8 weeks of an abrupt decrease in glycated hemoglobin-A1c (HbA1c). The underlying pathogenic mechanisms are still incompletely understood with only one mouse model being explored to date. The aim of this study was to further explore the hypothesis that an abrupt insulin-induced fall in HbA1c may be the prime causal factor of developing TIND. *BB/OKL* (bio breeding/*OKL*, Ottawa Karlsburg Leipzig) diabetic rats were randomized in three groups, receiving insulin treatment by implanted subcutaneous osmotic insulin pumps for 3 months, as follows: Group one received 2 units per day; group two 1 unit per day: and group three 1 unit per day in the first month, followed by 2 units per day in the last two months. We serially examined blood glucose and HbA1c levels, motor- and sensory/mixed afferent conduction velocities (mNCV and csNCV) and peripheral nerve morphology, including intraepidermal nerve fiber density and numbers of Iba-1 (ionized calcium binding adaptor molecule 1) positive macrophages in the sciatic nerve. Only in *BB/OKL* rats of group three, with a rapid decrease in HbA1c of more than 2%, did we find a significant decrease in mNCV in sciatic nerves (81% of initial values) after three months of treatment as compared to those group three rats with a less marked decrease in HbA1c <2% (mNCV 106% of initial values, *p* ≤ 0.01). A similar trend was observed for sensory/mixed afferent nerve conduction velocities: csNCV were reduced in *BB/OKL* rats with a rapid decrease in HbA1c >2% (csNCV 90% of initial values), compared to those rats with a mild decrease <2% (csNCV 112% of initial values, *p* ≤ 0.01). Moreover, *BB/OKL* rats of group three with a decrease in HbA1c >2% showed significantly greater infiltration of macrophages by about 50% (*p* ≤ 0.01) and a decreased amount of calcitonin gene related peptide (*CGRP*) positive nerve fibers as compared to the animals with a milder decrease in HbA1c. We conclude that a mild acute neuropathy with inflammatory components was induced in *BB/OKL* rats as a consequence of an abrupt decrease in HbA1c caused by high-dose insulin treatment. This experimentally induced neuropathy shares some features with TIND in humans and may be further explored in studies into the pathogenesis and treatment of TIND.

## 1. Introduction

Treatment induced neuropathy in diabetes (TIND) is seen as a severe, painful neuropathy, linked to an abrupt improvement in glycaemic control. Retrospective studies [1,2] have shown an acute and painful neuropathy in diabetic patients which manifested shortly after establishing control of diabetic hyperglycaemia. Gibbons and Freeman determined three criteria for diagnosis of TIND: an acute onset of neuropathic pain and/or autonomic dysfunction seen within 8 weeks after inducing an abrupt decrease in HbA1c of at least 2%. TIND is therefore seen as a mostly iatrogenic complication when treating diabetes [2,3]. It can afflict patients with type 1 or type 2 diabetes (T1D and T2D). The pathogenic mechanisms underlying TIND are widely unknown. In retrospective analyses, about 11% of patients experiencing a decrease in HbA1c of more than 2% over a period of 3 months developed TIND [2]. The type of antidiabetic therapy (e.g., use of insulin, oral hypoglycaemic agents or simply a rigorous diet) does not seem to be relevant. Treatment-induced neuropathy poses a conflict with the therapeutic goal of ameliorating not only hyperglycaemia but also the preventing treatment-induced sequelae such as diabetic induced neuropathy [3,4,5].

The pathogenesis of TIND is still incompletely understood [3,6]. In particular, suitable animal models seem to be lacking. A mouse TIND model has been previously described by Nicodemus et al. for type 2 diabetes mellitus [7]. TIND may occur in individuals with type 1 or type 2 diabetes treated with insulin and closely correlates with a rapid decrease in the glycated hemoglobin A1C [1,2]. The pathomechanism of diabetes in TIND mice with hyperinsulinemia and insulin insensitivity and *BB/OKL* (bio breeding/*OKL*, Ottawa Karlsburg Leipzig) rats with a lack of, or deficiency, of insulin seem to be completely different. Contrary to the higher-fat diet in the murine model, diabetes in *BB/OKL* rats develops by an autoimmune destruction of the insulin-producing pancreatic beta cells. Surprisingly, the observed decrease in HbA1c levels in insulin-treated *BB/OKL* rats, as important criterion of TIND, has been not evaluated in the murine TIND model [7].

In this study, an animal model for TIND was developed in *BB/OKL* rats. This strain is an established animal model for type 1 diabetes. *BB/OKL* rats spontaneously develop type 2 diabetes by an immune-mediated destruction of pancreatic β-cells, similar to human T1D. Hyperglycaemia generally begins between days 40 and 140 and is accompanied by weight loss, ketonuria, polyuria and dehydratation. Without insulin treatment *BB*-rats die within 5 and 10 days after manifestation of diabetes [8,9]. We hypothesized that this rat model may be utilized for further research into the pathogenesis of TIND.

## 2. Results

### 2.1. Effect of Different Modes of Insulin Tretment on HbA1c and Blood Glucose in BB/OKL Diabetic Rats

**Figure 1:**
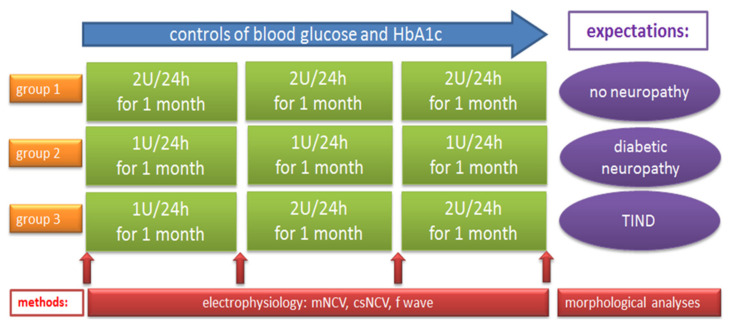
Study design showing the three groups of *BB/OKL* (bio breeding/*OKL*, Ottawa Karlsburg Leipzig) rats with different insulin therapy over 3 months and the performed analyses. Male (*n* = 38), 2-month old, *BB/OKL* rats were randomized into three groups receiving insulin treatment by implanted subcutaneous osmotic insulin pumps. Group 1 animals received 2U of insulin and group 2 animals 1U of insulin per 24 h for 3 months. Group 3 animals received 1U per 24 h in the first month, followed by 2U per 24 h in the second and third month. mNCV = motor nerve conduction velocity, csNCV = combined sensorimotor nerve conduction velocity, U = unit, TIND = treatment-induced neuropathy in diabetes.

The three groups of *BB/OKL* diabetic rats received insulin treatment by implanted subcutaneous osmotic insulin pumps for 3 months. Group one (*n* = 12) received 2 units (U) per day; group two (*n* = 10) 1 unit per day, whereas group three (*n* = 16) received 1 unit per day in the first month, followed by 2 units per day in the second and third months (Figure 1).

**Figure 2:**
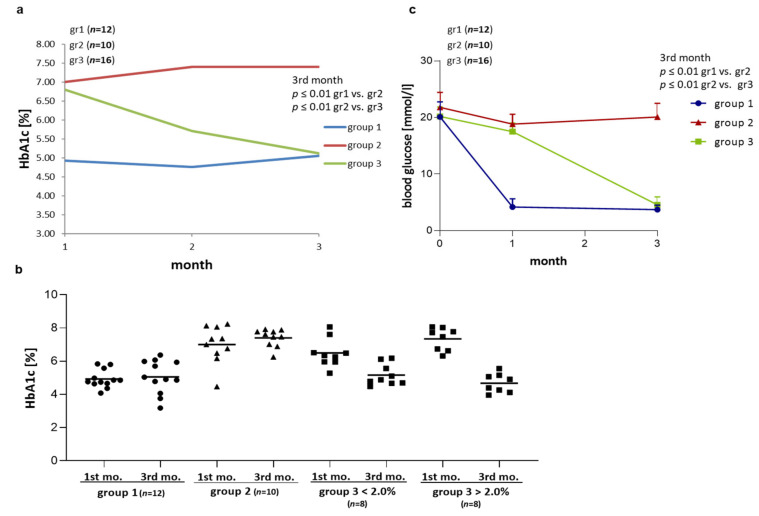
Effect of insulin treatment on blood glucose concentrations and hemoglobin-A1c (HbA1c) levels. (**a**) HbA1c values over time in the three groups of *BB/OKL* rats (mean HbA1c values ± SD are given in Table 2). (**b**) Dot plot represents HbA1c values at the end of month 1 and 3 in all experimental groups. Group 3 has been stratified by decrease in hemoglobin-A1c. (**c**) Blood glucose concentration (mean values ± SD are given in Table 1). A blood glucose concentration of ≥16 mmol/L in *BB/OKL* rats was a criterion to include the animals in the studies. Note that 2U of insulin treatment in group 1 (*n* = 12) and in group 3 animals (*n* = 16; after 2nd month) significantly decreased blood glucose and HbA1c levels compared with 1U of insulin treated animals of group 2 (*n* = 10).

The insulin treatment significantly decreased blood glucose concentrations in BB/OKL rats at the end of month 3 as compared with starting concentrations: in group one from 20.8 ± 2.5 to 4.1 ± 0.7 and in group three from 20.3 ± 2.1 to 4.3 ± 0.7 (*p* ≤ 0.01). The treatment with 1 unit in group two resulted in ongoing hyperglycaemia between 0–1 month (21.4 ± 2.5) and at the end of month 3 (20.2 ± 2.5) of the experiment (Table 1 and Figure 2c).

As expected, HbA1c levels differed between the three groups (Table 2 and Figure 2a,b). Group one (2 units of insulin) had stable average HbA1c values around 5%, indicating successful induction of stable euglycaemia. Group two (1 unit of insulin) showed stable and higher average HbA1c values of around 7–8% over three months of consistent hyperglycaemia. The animals in group three showed average HbA1c levels of 6.8% after the first month under treatment with 1 U insulin per day. The increase in the dose to 2 U per day reduced the HbA1c levels to an average of 5.7% in the second month and to 5.1% at the end of month 3. Therefore, in group three this treatment schedule induced the intended abrupt decrease in HbA1c upon doubling the insulin dose. This variability is reflected in the range of HbA1c reductions in group three, when comparing the end of the first and the third month: i.e., minus 0.87 to plus 3.21% with negative values indicating even a slight increase in HbA1c at the end of month 3. More specifically, eight of the 19 rats in group three reached a HbA1c reduction of more than 2%, while 11 of 19 showed milder reductions of less than 2% or even showed a small increase.

### 2.2. Effect of Different Modes of Insulin Treatment on Nerve Conduction Velocities, F Waves and Amplitudes of Sciatic Nerve in BB/OKL Diabetic Rats

**Figure 3:**
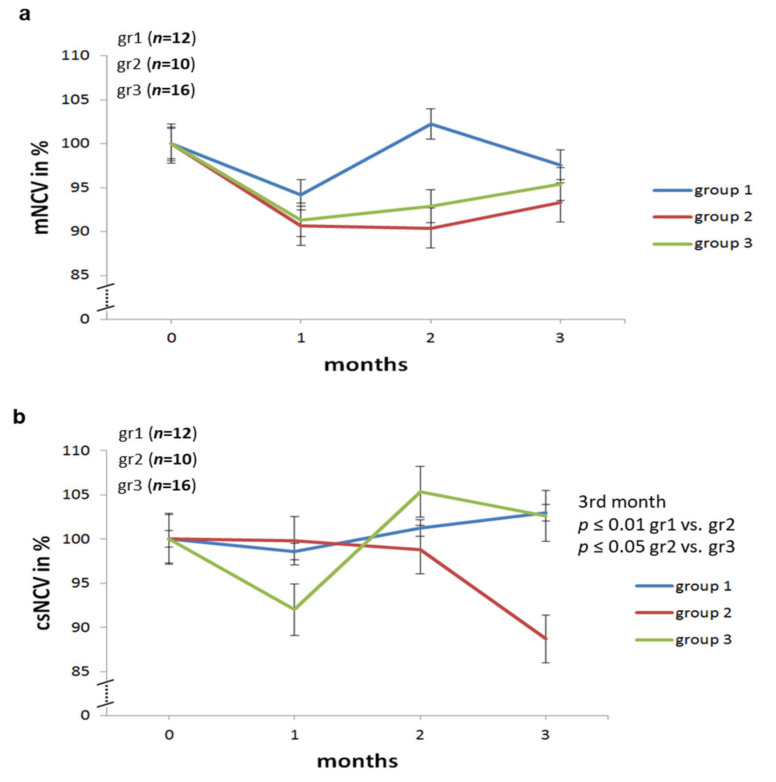
Normalized mNCV (motor nerve conduction velocity) and csNCV (combined sesorimotor nerve conduction velocity) values in percent of the initial values for each time point in sciatic nerve of *BB/OKL* rats in three groups. (**a**) No significant differences in motor NCV were found between animal groups. (**b**) The sensory/mixed afferent NCV was significantly reduced in group 2 rats (*n* = 10) vs. group 1 (*n* = 12) and group 3 (*n* = 16) rats (mean values ± SD are given in Table 3).

As shown in previous studies [10,11,12], individual nerve conduction velocities can vary widely in rats, even in the absence pathologic conditions such as diabetes. For this reason, we normalized nerve conduction velocities as well as the minimum F wave latency time as a percentage of the initial value set at 100%. This means that for each individual rat electrophysiological results of the one-, two- and three-month-measurements were normalized to the baseline. This allowed us to calculate the relative changes over time from the beginning, i.e., prior to any onset of diabetes, up to the end of the experimental period. Motor nerve conduction velocities at baseline did not differ significantly between the three groups (Table 3 and Figure 3). Specifically, motor nerve conduction velocities in all of group three, irrespective of HbA1c values, were not significantly different from those of group one or group two.

Mixed, afferent nerve conduction velocities did not differ between groups. In group three, the combined sensorimotor nerve conduction velocity (csNVC) decreased only initially under low dose insulin therapy. However, there was no significant decrease in csNCV at the last test. In contrast, group two showed a distinct decrease in csNCV after three months compared to the other two groups (Table 3 and Figure 3) showing that inducing functional pathology took longer under insufficient insulin treatment.

In evaluating F waves, we focused on the minimum F wave latency in ten serial stimulations, as this parameter is also in humans is used as an additional parameter in the assessment of nerve dysfunction. No significant differences were found between the three groups (Table 3). The same applied to other parameters like F wave persistence. Moreover, no differences were found either in the CMAP (compound muscle action potential) amplitudes (elicited by motor nerve stimulation) nor in the CSNAP (compound sensory nerve action potential) amplitudes (elicited by distal stimulation).

### 2.3. Nerve Conduction Studies Stratified by the Decrease in HbA1c Levels in Group 3 of BB/OKL Rats

**Figure 4:**
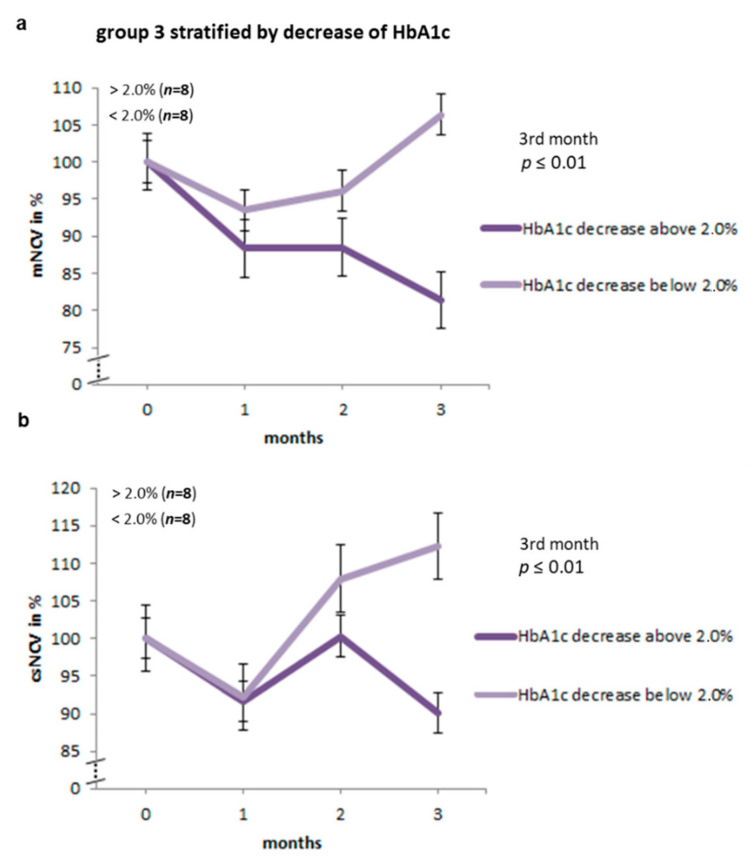
Normalized mNCV (motor nerve conduction velocity) and csNCV (combined sesorimotor nerve conduction velocity) values in animals of group 3 stratified by decrease in HbA1c and depicted for each time point in percent of the initial values. Note that *BB/OKL* rats with a decrease in HbA1c of more than 2% showed significant slower mNCV (**a**) and scNCV (**b**) after three months as compared to the animals with a decrease of less than 2% (mean values are given in Table 4). Data from *n* = 8 per group are presented as mean ± SD.

As outlined above group results showed no significant differences in electrophysiological assessments and more specifically, group three did not, as a whole, exhibit a more marked neuropathy. Experimental animals in group three differed markedly in their individual response to the different treatment regimes. Therefore, in a subsequent exploratory analysis, we stratified animals of group three according to their magnitude of therapeutically induced decrease in HbA1c levels between the first and the third month. Rats with a decrease in HbA1c of more than 2% showed significantly slower motor nerve conduction velocity (mNCV) after three months (minimum 60.3%, maximum 99.6%, mean 81.4%) as compared to the animals with a decrease of less than 2% (minimum 68.3%, maximum 167.6%, mean 106.4%; *p* ≤ 0.01; Table 4 and Figure 4). Similarly, the csNCVs were significantly reduced in group three rats with a larger drop of HbA1c as compared with rats exhibiting a smaller or no drop of HbA1c after three months (rats with a decrease in HbA1c >2%: minimum 66.6%, maximum 145.9%, mean 90.1%; rats with decrease in HbA1c <2%: minimum 87.8%, maximum 187.1%, mean 112.3%; *p* ≤ 0.01; Table 4 and Figure 4). No differences could be observed in minimum F wave latencies (Table 4).

### 2.4. Infiltration of Inflammatory Cells in Sciatic Nerves of BB/OKL Rats after Different Modes of Insulin Treatment

**Figure 5:**
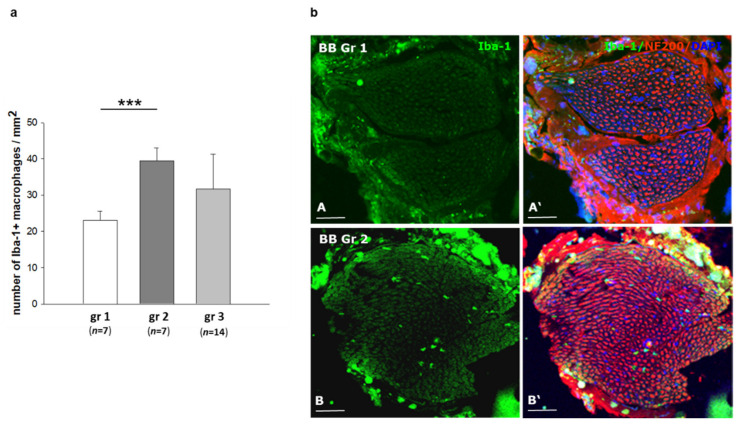
Macrophage distribution in cross sections of sciatic nerves from *BB/OKL* rats at the end of month 3. (**a**) Quantification of *Iba-1* (ionized calcium binding adaptor molecule 1) —positive macrophages. (**b**) Double IF (immunofluorescence) staining for *Iba-1* (green, macrophages) and for neurofilament 200 (red, nerve fibers). Note that immunoreactivity for *Iba-1* positive macrophages was higher in sciatic nerves of *BB/OKL* rats in the group 2 (B) as compared to group 1 (A). Nuclei are stained with DAPI (4′,6-diamidino-2-phenylindol, blue). Bars represent 100 µm (AB,A’B’). Data from *n* = 7 per group are presented as mean ±SEM. *** *p* ≤ 0.001 according to the one-way analysis of variance together with the Newman–Keuls test.

**Figure 6:**
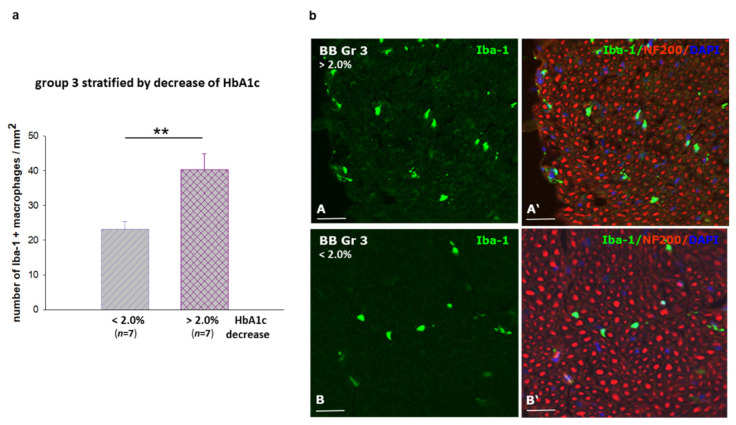
Macrophage distribution in sciatic nerves of *BB/OKL* rats in group 3 stratified by the reduction in HbA1c values at the end of month 3. (**a**) Quantification of *Iba-1* (ionized calcium binding adaptor molecule 1)—positive macrophages. Data from *n* = 7 are presented as mean ±SEM. ** *p* ≤ 0.01 according to the one-way analysis of variance together with the Newman–Keuls test. (**b**) Representative photomicrograph of double IF staining for *Iba-1* (green, macrophages) and for neurofilament 200 (red). Note that the greater number of macrophages in sciatic nerve correlated with a larger reduction of HbA1c values >2% as compared to the lesser reduction in HbA1c values <2%. Nuclei are stained with DAPI (4′,6-diamidino-2-phenylindol, blue). Bars represent 50 µm (AB–A’B’).

The peripheral neuropathy from mild to acute form is generally characterized by an infiltration of macrophages into the peripheral nerve [13,14]. We therefore investigated a potential inflammatory component by immunostaining against the microglia/macrophage ionized calcium binding adaptor molecule 1 (*Iba-1*) to identify macrophages in sciatic nerves of the *BB/OKL* rats in all experimental groups. We found infiltration of macrophages to be about 50% less in nerves of group one rats compared to groups two and three (Figure 5a,b). More importantly, in group three rats the number of inflammatory cells correlated with the magnitude of the decrease in HbA1c values. In nerves from rats with a larger reduction in HbA1c values, i.e., >2%, infiltration of macrophages was significantly more pronounced as compared to the animals with a smaller decrease in HbA1c values, i.e., <2% (Figure 6a,b).

### 2.5. Effect of Different Modes of Insulin Treatment on Intraepidermal Nerve Fiber Density (IENFD) and Calcitonin Gene Related Peptide CGRP Expression in Skin Speciments of BB/OKL Rats

**Figure 7:**
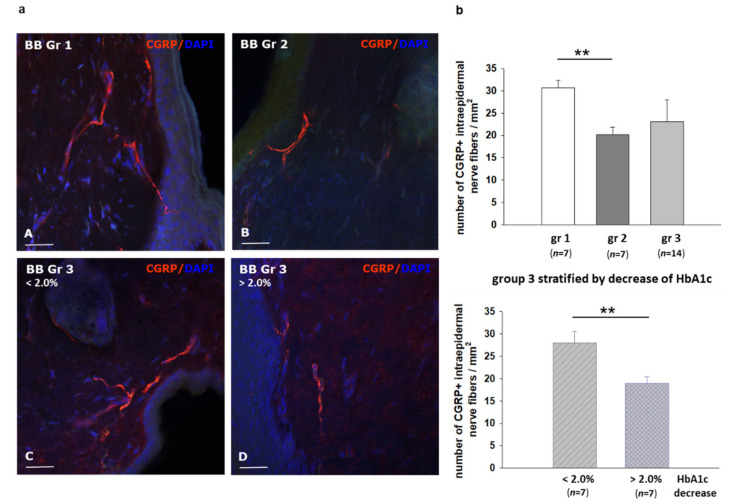
Sensory nerve endings in the skin of the hind foot in *BB/OKL* rats at the end of month 3. (**a**) Immunofluorescence staining for calcitonin gene related peptide (*CGRP*) protein (red, A-D). Nuclei are counterstained with DAPI (4′,6-diamidino-2-phenylindol, blue). (**b**) Quantification of *CGRP*—positive IENFDs (intraepidermal nerve fiber density) in group 3 separated by the reduction in HbA1c values. A marked reduction in *CGRP* expression was noted in subgroup 3 with a reduction in HbA1c values >2%. Data from *n* = 7 are presented as mean ±SEM. ** *p* ≤ 0.01 according to the one-way analysis of variance together with the Newman–Keuls test. A-D: Bars represent 15 µm.

Intraepidermal nerve fiber density (IEDNF) was determined by quantitative immunofluorescence on skin specimens of *BB/OKL* rats using *PGP 9.5* (protein gene product 9.5) antibody and sensory nerve endings were detected with *CGRP* (calcitonin gene related peptide) iimmunostaining. A total of 75 sections from the ten skin specimens were analyzed. No significant difference in IENFD was found between the three groups (Supplementary Figure S1a). IENFD did not differ in group three separated by the decrease in HbA1c values (HbA1c >2%: 27 ± 2.3 fibers/mm^2^ and HbA1c <2%: 25 ± 2.1 fibers/mm^2^; Supplementary Figure S1b). However, following insulin treatment, a significant reduction in the number of the *CGRP*-positive fibers was observed in group 2 nerves (1U insulin) in comparison to group 1 (2 U insulin; 20 ± 3.1 versus 31 ± 2.0 fibers/mm^2^). Moreover, in the group 3, we detected a statistically significant decrease in the *CGRP* expression, when comparing the rats with a higher versus lower decrease in HbA1c values (HbA1c reduction >2%: 19 ± 1.4 fibers/mm^2^ versus HbA1c reduction <2%: 28 ± 1.8 fibers/mm^2^) (Figure 7). This may indicate that *CGRP* expression correlates with the degree of TIND-like pathology.

## 3. Discussion

Stimulated by previous retrospective studies in patients with TIND showing a strong correlation between the risk of developing TIND and the magnitude of decrease in HbA1c after diabetes therapy [1,2], we addressed the hypothesis that a rapid fall in HbA1c levels may be a causal factor in inducing TIND. To do this, we aimed at generating an animal model for TIND in a rat strain prone to spontaneously develop type 1 diabetes mellitus.

Our principal finding in the present study corroborates the hypothesis that the rapid fall in HbA1c upon insulin-induced normoglycaemia may be the main causative factor of TIND in type 1 diabetes. We found a significantly higher decrease in motor and compound sensory nerve conduction velocities after three months of insulin treatment in *BB/OKL* rats with a decrease in HbA1c of more than 2%, compared to those with a decrease of less than 2% in group three. A higher decrease in mNCVs and scNCVs was associated with evidence of an enhanced inflammatory response. Similar to observations in diabetic patients, the response of diabetic rats to equivalent insulin doses appears to vary considerably, resulting in a variable treatment response, as indicated by HbA1c levels. Therefore, it seems likely that the antidiabetic therapy itself may not be the sole factor responsible in developing TIND but rather the individual metabolic response to treatment may be instrumental. Indeed, it is surprising that rats sharing the same genetic background, same diet and insulin treatment regimen exhibit such a high degree of variation in the HbA1c response. We cannot exclude that factors such as differences in food intake or absorption, metabolic rate, spontaneous activity or individual insulin treatment response may underly the heterogenous HbA1c effects in group three. In addition, heterogeneity in the incidence of diabetes has been described for this rat model [15] and variation in insulin treatment responses has been reported for similar animal models despite the same genetic background [16,17].

In the rat model used here, a sharp decrease in HbA1c seems to be the principal marker associated with the risk of developing TIND. There are few reports on further pathogenic mechanisms of TIND. Regarding the correlation with an abrupt change in glycaemic metabolism Low and Singer [6] proposed a concept of “energy crisis”: in animal models, chronic hyperglycemia leads to an increased intercapillary distance, reduced nerve blood flow and endoneural oedema, resulting in a long-standing hypoxic endoneural microenvironment [18]. Due to the proposed poor blood flow autoregulation in peripheral nerves, nerve fibers may be susceptible to metabolic milieu changes [6,18]. Conceivably, an abrupt drop in glucose levels could cause a relative endoneural hypoglycaemia, leading to an “energy crisis”. This mechanism has been hypothesized to underlie deterioration of neuropathy in children with T1D [19,20,21] Supporting this hypothesis, Mohseni and co-authors showed that hypoglycaemia episodes induced progressive neuropathic changes in insulin-treated diabetic rats [22]. This theory would link development of TIND to abrupt decreases in HbA1c. Further studies with an explicit focus on the pathogenetic mechanisms of TIND taking hypoglycaemia into account are required.

TIND is diagnosed as a painful neuropathy in which the pain is difficult to be controlled by medical treatment. The majority of diabetic patients develop neuropathic pain which is resistant to currently available medications [23,24,25]. Chronic inflammation in response to an infiltration of macrophages into peripheral nerves has been reported to play a pivotal role [26,27,28]. Within the macrophage-derived cytokines, *IL-1*, *IL-6* and tumor necrosis factor alpha (*TNF-α*) belong to neuro-inflammatory regulators that enhance the excitability of primary sensory neurons and trigger neuropathic pain [29,30,31,32]. Notably, the inhibition of macrophages results in the prevention of experimental neuropathic pain in rodents [27,33,34].

Our results showed a significant reduction in macrophages of about 50% in sciatic nerves of *BB/OKL* rats after optimal insulin treatment compared with animals from other experimental groups. However, the analysis of group three animals stratified by the decrease in HbA1c >2% showed significantly greater infiltration of macrophages as compared to the animals with a decrease of <2%. The more pronounced infiltration of macrophages, accompanied by the slower mNCV and csNCV in the rats with rapid decrease in HbA1c, may indicate an acute and potentially pathogenic inflammatory component in this rat model. Therefore, we speculate that this pathogenic pathway in the *BB/OKL* rats might also play a role in TIND in humans. Notably, this apparent numeric ‘threshold‘ of HbA1c corresponds to the value found in human diabetic patients [2,35].

Previously it was assumed that TIND primarily affects thinly myelinated Aδ-fibers and unmyelinated C-fibers [1,34]. In view of the “energy crisis” concept [6], the unmyelinated fibers have a larger surface to size ratio making them considerably more susceptible to such a loss of glucose or ATP. In our study, the abnormalities in nerve conduction studies revealed that larger myelinated nerve fibers of the sciatic nerve (including Aα- and Aβ-fibers) were also affected. These findings cannot be compared to human TIND as yet, because in human patients electroneurography has not been comprehensively performed [1,2,35].

However, the dysfunction of peripheral nerves in the present study was not associated with significant morphological degenerative changes in IENFD (Supplementary Figure S1), the expression of calcitonin gene related peptide was significantly reduced (Figure 7). This may point to a neuropathic pain state since *CGRP* neuropeptide is synthesized and released by nociceptive sensory neurons [36].

Besides this theory, there are different findings concerning the role of nerve degeneration und regeneration. While previous studies confirmed a close correlation between nerve degeneration and neuropathic pain, there are also findings that nerve regeneration plays an important role [37,38]. Spontaneously regenerating nerve fibers could be an additional cause of neuropathic pain.

In conclusion, the manifestation of TIND induced by insulin treatment in *BB/OKL* rats has been linked to the magnitude and timing of a marked reduction in HbA1c, as was suggested in patients with type 1 diabetes mellitus [2,35,39]. Our results support the notion that strong and rapid optimization of chronic hyperglycaemia may contribute to TIND [3,39]. More experimental research is warranted to further elucidate the various potential pathways ultimately leading to this enigmatic complication of type 1 and type 2 diabetes.

## 4. Material and Methods

### 4.1. Animals and Treatments

We included a total of 65 male, 2-month old, *BB/OKL* rats (bio breeding/*OKL*, Ottawa Karlsurg Leipzig) of which 27 either never experienced manifestation of diabetes or died early for unidentified reasons. These were excluded from further analyses. The remaining 38, 2-month old *BB/OKL* male rats were randomized as to allocation of the treatment groups, 12 in group 1, 10 in group 2 and 16 in group 3. All rats received the same standardized pallets diet and water ad libitum.

Immediately after onset of hyperglycemia, subcutaneous ALZET osmotic insulin pumps (Charles River Laboratories, Germany GmbH, Sulzfeld, Germany) were implanted and animals were randomized into three treatment groups—differing in the degree of glycaemic control and in the absence and presence of a sharp transient in establishing euglycemia. Group 1 animals received 2 units (U) of insulin per 24 h for 3 months, a treatment intended to sufficiently control diabetes and maintain euglycemia throughout the entire experimental period with HbA1c levels expected to stay at around 4–5%. Group 2 animals received 1U per 24 h for 3 months, a treatment intended to insufficiently control diabetes and maintain hyperglycemia, with HbA1c levels expected to stay at around 7–8%. Group 3 animals received 1U per 24 h in the first month, followed by 2U per 24 h in the second and third month. The intention of this schedule was to induce an abrupt decrease in HbA1c between the first month of treatment and the following two months. We hypothesized that group 3 animals would be at a high risk of developing TIND, whereas group 1 animals and group 2 animals served as different control groups (Figure 1). To detect functional and morphological pathology in peripheral nerves, electrophysiological (electroneurography) and neuropathological examinations including immunohistochemical analyses were performed. Additionally, general information (weight, blood glucose, HbA1c) was acquired at predefined intervals.

Experiments followed the international guidelines of animal care and the study protocols were approved by the Landesdirektion Leipzig, the local authority for animal care (approval ID: TVV 04/16).

### 4.2. Electroneurography

Electrophysiological measurements of nerve conduction velocity were employed as a functional well established method to examine and quantify peripheral nerve pathology in mouse or rat disease models [9,10,11]. Electroneurography was performed on each rat prior to onset of hyperglycemia, and after the first, second and third month of insulin treatment. The measurements were performed on the sciatic nerve under inhalation anesthesia (isoflurane 2.5%). To determine motor nerve conduction velocity (mNCV) supramaximal stimulation was applied at the distal tibial nerve and at the fibular nerve in the region of the ankle joint, followed by a supramaximal stimulation proximal at the sciatic notch. Near-nerve position of the proximal electrodes was ascertained by a threshold of less than 5 mA. Bare steel needle electrodes were used distally and near nerve insulated tungsten electrodes proximally (original DISA electrodes) for all measurements. The compound muscle action potentials (CAMPs) were recorded with steel electrodes between digits 2 and 3 (active electrode) and digit 5 (reference electrode). Motor nerve conduction velocity was then calculated as the ratio of the latency difference in millimetres between distal and proximal stimulations divided by the distance between the stimulation electrodes. The amplitudes of the CMAPs were measured. Additionally, F waves (late potentials) were recorded upon 10 serial stimuli at 1.5 per second, and the shortest latencies were measured.

To examine the compound sensory and motor compound nerve conduction velocity (csNCV) stimulation took place at the ankle joint with recording the compound nerve action potential at the sciatic notch. csNCV was then calculated as a ratio of latency over the distance between electrodes. The amplitudes of the compound sensory nerve action potentials (csNAP) were also recorded.

### 4.3. Immunostaining

*BB/OKL* rats (*n* = 7 per group) were perfused via the left heart ventricle with 4% formaldehyde in 0.1 M PBS. The sciatic nerves and food skin specimens were postfixed in the same fixative for 4 h, rinsed with PBS and transferred into 30% buffered sucrose solution and frozen. The frozen cross sections of sciatic nerves and foot skin specimens were mounted on gelatinized glass slides. For detection of macrophages the rabbit polyclonal microglia/macrophage cytoplasmatic calcium adaptor (*Iba-1)* antibody (1:300; WAKO Chemicals USA, Richmond, VA, USA) was used for double staining with the mouse monoclonal antibody against neurofilament 200 (*NF200*; 1:500; Sigma Aldrich, Taufkirchen, Germany). The intraepidermal small nerve fibers were detected with polyclonal antibody against protein gene product (*PGP*) 9.5 and monoclonal antibody against *CGRP* (both 1:1000; Abcam, MA, USA). *Cy3* (cyanin)-conjugated goat anti-mouse IgG and *FITC* (fluorescein isothiocyanate)-conjugated goat anti-rabbit IgG were used as secondary antibodies (diluted 1:1000; Dianova, Hamburg, Germany). Nuclear staining was performed with 10 μg/mL DAPI (Serva, Heidelberg, Germany). Immunostaining without the primary antibody served as negative controls (Supplementary Figure S2; A: cross-section of sciatic nerve; B: cross section of the skin specimen).

### 4.4. Determination of Macrophages

A total of 63 sciatic nerve cross sections (from *n* = 7 *BB/OKL* rats each per animal groups 1, 2, and 3) were used to determine the number of *Iba-1* positive macrophages per mm^2^ as described previously [11].

### 4.5. Quantification of the Intraepidermal Nerve Fiber Density (IENFD) and CGRP-Positive Nerve Endings

The skin sections from the hind foot of *BB/OKL* rats (from *n* = 7 *BB/OKL* rats each per groups 1, 2, and 3) were analyzed for determining the number of *PGP 9.5* (protein gene product 9.5) and *CGRP* (calcitonin gene related peptide) positive intraepidermal nerve fibers crossing the dermal-epidermal junction and from individual fibers in the dermis and epidermis as described previously [11].

### 4.6. Statistical Analyses

Data are presented as means ± SD (Table 1 and Table 2; Figure 2, Figure 3 and Figure 4) or as means ± SEM (Figure 5, Figure 6 and Figure 7). Differences among the experimental groups were calculated using one-way-ANOVA followed by the Newman-Keuls test (SigmaStat; San Rafael, CA, USA). *p*-values < 0.05 were considered as statistically significant.

## 5. Conclusions

By treating diabetic *BB/OKL* rats with an insulin dose, causing an abrupt decrease in HbA1c levels, we were able to induce an acute form of neuropathy. This may be considered as an animal model of TIND equivalent to TIND in human type 1 diabetes. A comprehensive further investigation may help to elucidate the potentially crucial factors in the pathogenesis of TIND and ultimately aid the discovery of preventive measures.

## Figures and Tables

**Table 1:** Mean blood glucose concentrations in the three experimental groups of rats. Data from group 1, *n* = 12; group 2, *n* = 10; group 3, *n* = 16 are presented as means ±SD.

Blood Glucose (mmol/L) ±SD	0 Month	After 1st Month	After 3rd Month
group 1	20.8 ± 2.5	3.9 ± 1.0	4.1 ± 0.7
group 2	21.4 ± 2.4	18.8 ± 1.7	20.2 ± 2.4
group 3	20.3 ± 2.1	19.1 ± 1.9	4.3 ± 1.2

**Table 2:** Mean HbA1c for every measurement point in the three groups of rats (group 1, *n* = 12; group 2, *n* = 10; group 3, *n* = 16).

HbA1C (%) ±SD	After 1st Month	After 2nd Month	After 3rd Month
group 1	4.9 ± 0.9	4.8 ± 1.5	5.1 ± 0.8
group 2	7.0 ± 1.1	7.4 ± 0.8	7.4 ± 0.6
group 3	6.8 ± 1.2	5.7 ± 1.3	5.1 ± 1.0

**Table 3:** Averaged values of mNCV (motor nerve conduction velocity), csNCV (combined sesorimotor nerve conduction velocity) and minimum F wave latencies for the three rat groups (group 1, *n* = 12; group 2, *n* = 10; group 3, *n* = 16) over three months shown as normalized to 100% of the initial value.

		Start	After 1st Month	After 2nd Month	After 3rd Month
group 1	mNCV	100	94.2	102.2	97.6
	csNCV	100	98.6	101.2	103
	F wave	100	95	93	92
group 2	mNCV	100	90.7	90.4	93.3
	csNCV	100	99.8	98.8	88.7
	F wave	100	101	94.3	99
group 3	mNCV	100	91.3	93	95.4
	csNCV	100	92	105.4	102.6
	F wave	100	98.5	95	91.4

**Table 4:** Averaged values of mNCV (motor nerve conduction velocity), csNCV (combined sesorimotor nerve conduction velocity) and minimum F wave latencies for group 3 over three months stratified by the decrease in HbA1c levels (>2%, *n* = 8; <2%, *n* = 8) each shown as normalized to 100% of the initial value.

		Start	After 1st Month	After 2nd Month	After 3rd Month
decrease in HbA1c >2%	mNCV	100	88.3	88.5	81.3
	csNCV	100	91.7	100.3	90
	F wave	100	95.6	92	88
decrease in HbA1c <2%	mNCV	100	93.5	96	106.6
	csNCV	100	92.2	108	112.3
	F wave	100	100.5	97.2	94

## Data Availability

The data presented in this study are available in this manuscript.

## Supplementary Materials

The following are available online at https://www.mdpi.com/1422-0067/22/4/1571/s1, Figure S1: Quantification of *PGP 9.5* (protein gene product 9.5)—positive IENFDs (intraepidermal nerve fiber densities) in the skin of the hind foot in *BB/OKL* rats at the end of month 3. Figure S2: Negative controls for immunostaining without the primary antibodies (A: cross-section of sciatic nerve; B: cross section of the skin specimen).
