# Supplementary figures and images for: Treatment-Induced Neuropathy in Diabetes (TIND)—Developing a Disease Model in Type 1 Diabetic Rats
Source: Int J Mol Sci. 2021 Feb 4;22(4):1571. doi: 10.3390/ijms22041571 (PMC7913916; doi:10.3390/ijms22041571)

**a**

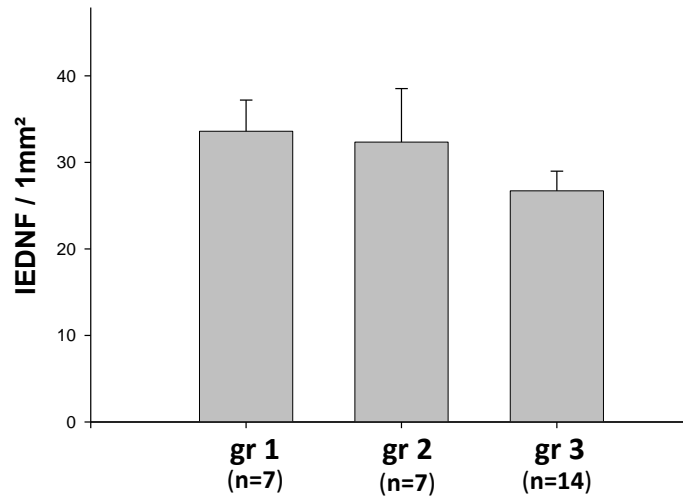

**b**

**group 3 stratified by decrease of HbA1c**

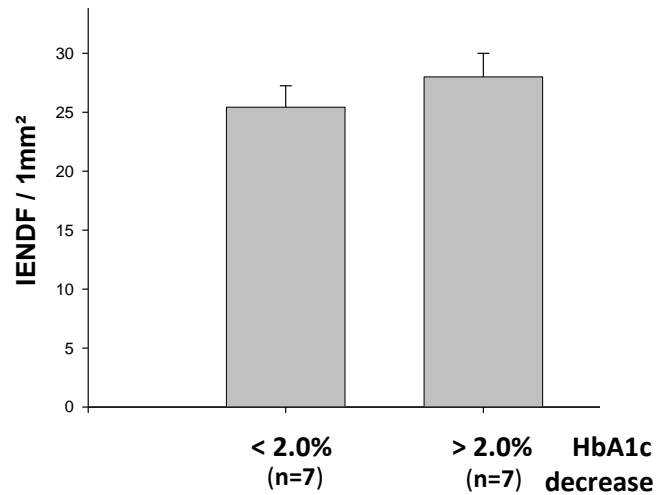

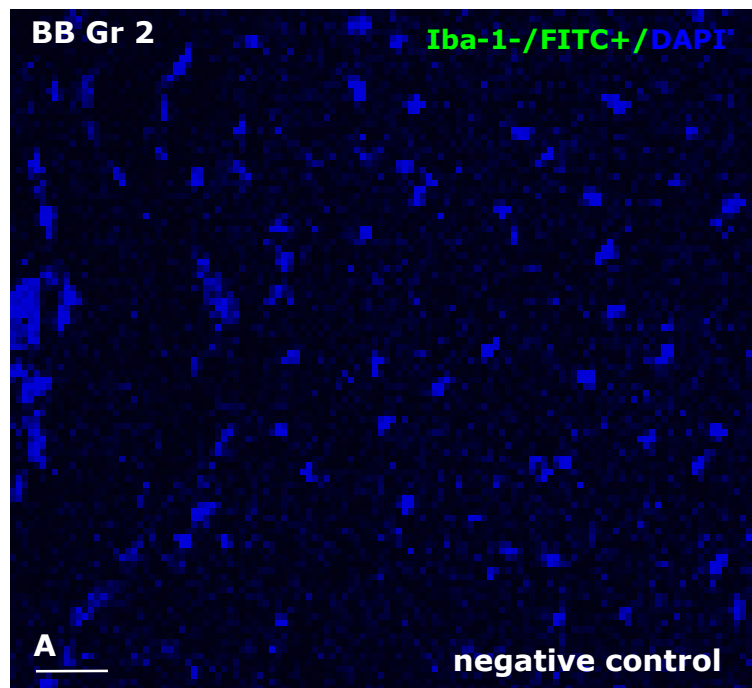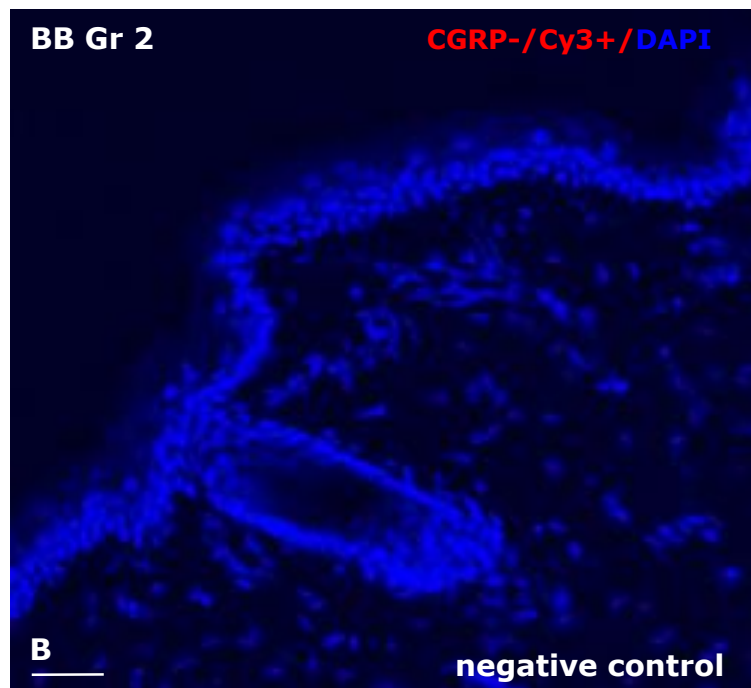

Supplement: Supplementary file 1 [file ijms-22-01571-s001.pdf]
